# Comparison of the effect of QMix and conventional root canal irrigants on push‐out bond strength of fiber post to root dentin

**DOI:** 10.1002/cre2.500

**Published:** 2021-10-18

**Authors:** Farzaneh Afkhami, Mona Sadegh, Aidin Sooratgar, Maryam Amirmoezi

**Affiliations:** ^1^ Department of Endodontics School of Dentistry, Tehran University of Medical Sciences, International Campus Tehran Iran; ^2^ Endodontist, Private Practice Vancouver British Columbia Canada; ^3^ General Dentist, Private Practice Tehran Iran

**Keywords:** fiber post, final rinse, push‐out bond strength, QMix

## Abstract

**Objectives:**

This study aimed to assess the effect of application of QMix and common root canal irrigating solutions on the bond strength of fiber post to root dentin.

**Material and Methods:**

In this in‐vitro study, 60 extracted incisor teeth were decoronated such that 15 mm of root length remained. The canals were prepared with ProTaper rotary system to F5 and filled with gutta‐percha and AH26 sealer. Prior to post placement, the teeth were divided into four groups based on the type of final irrigating solution namely saline, 5.25% NaOCl, 2% chlorhexidine, and QMix. The fiber posts were then cemented with Panavia F2 resin cement. The roots were sectioned perpendicular to their longitudinal axis, and four sections with 1 mm thickness were made at the middle and coronal thirds of the roots. The push‐out bond strength of fiber posts was measured by a universal testing machine in megapascals. Data were analyzed by two‐way ANOVA and Tukey's test.

**Results:**

The maximum and minimum bond strength values were noted in QMix and NaOCl groups in both the middle and coronal third of the root, respectively. But, there was no significant difference between the push‐out bond strength in the middle or coronal third of the root (*p* = 0.054). Adhesive failure was the most common mode of failure in all groups.

**Conclusion:**

Use of QMix for post space irrigation does not compromise the bond strength of fiber post to root dentin, and can be used for final rinsing of the post space.

## 1 INTRODUCTION

Fiber posts were introduced as an alternative to cast post and cores and metal dowels for restoration of endodontically treated teeth that have lost a great portion of their structure. These posts have advantages such as higher esthetics (Zicari et al., 2008). Also, they have a modulus of elasticity similar to that of dentin and therefore, significantly decrease the risk of vertical root fracture (Zicari et al., 2008).

Sodium hypochlorite (NaOCl) has long been used as one of the most common root canal irrigating solutions (Stojicic et al., 2012). Its popularity is attributed to its bactericidal and virucidal properties as well as its optimal tissue dissolution ability (Khalilak et al., 2011). Moreover, NaOCl has a low viscosity and long shelf‐life (Stojicic et al., 2012). On the other hand, NaOCl is incapable of elimination of the smear layer and therefore, it is often used along with a chelating agent such as ethylenediaminetetraacetic acid (EDTA) (Dibaji, Afkhami, et al., 2017; Gündoğar et al., 2018). Use of NaOCl as final root canal irrigant after EDTA may compromise the structural integrity of root dentin (Gündoğar et al., 2018). Also, evidence shows that use of NaOCl for final irrigation can decrease the bond strength of sealers and glass fiber posts to root dentin (Elnaghy, 2014; Gündoğar et al., 2018).

Chlorhexidine gluconate (CHX) is also commonly used as a root canal irrigating solution and an intracanal medicament due to its antimicrobial activity, long‐term substantivity, and low cytotoxicity (Gündoğar et al., 2018). One advantage of CHX as root canal irrigating solution is that it does not compromise the bond strength of resin to root dentin (Afkhami et al., 2018). Nonetheless, evidence shows that NaOCl is superior to CHX in terms of antimicrobial activity and tissue dissolution ability (Afkhami et al., 2018). CHX cannot dissolve the mineral phase of the smear layer either (Gündoğar et al., 2018). Also, if used in combination with NaOCl, it results in formation of a toxic precipitate known as parachloroaniline in the root canal system, which negatively affects the root canal seal by the root filling materials (Gündoğar et al., 2018).

QMix is a new root canal irrigant for elimination of the smear layer, which has antimicrobial properties as well (Gündoğar et al., 2018). It contains EDTA, CHX, a detergent, and deionized water (Elnaghy, 2014). It was introduced as a final root canal irrigating solution, and should be used for 60 to 90 s according to the manufacturer's instructions. It is a ready‐to‐use clear solution and evidence shows that it can effectively remove the smear layer and bacteria such as *Enterococcus faecalis* (Elnaghy, 2014; Gündoğar et al., 2018). QMix solution increases the radicular dentin demineralization due to the chelating effects of EDTA and simultaneous disinfecting properties (Dai et al., 2011; Stojicic et al., 2012). The main reason behind addition of surfactant to QMix is to decrease the surface tension of the solution and simultaneously increase the wettability and flowability of the irrigating solution in the root canal system to enhance its contact with the smear layer and the underlying dentin (Giardino et al., 2006; Stojicic et al., 2012). These observations have been confirmed by scanning electron microscopic studies as well, and the optimal efficacy of QMix for elimination of the smear layer has been well documented (Elnaghy, 2014).

Considering the necessity of root canal disinfection prior to cementation of prefabricated posts, and the possible effects of root canal irrigating and disinfecting solutions on bond strength of fiber posts to root dentin, this in vitro study aimed to compare the effects of QMix and conventional root canal irrigating solutions (NaOCl, CHX, and saline) on bond strength of fiber post to root dentin.

## 2 MATERIALS AND METHODS

This study has been submitted to the research ethics committee of Tehran University of Medical Sciences (IR.TUMS.DENTISTRY.REC.1395.88) and the study was performed in vitro on human teeth extracted for orthodontic or periodontal reasons after obtaining informed consent of patients.

This in vitro experimental study evaluated 60 sound extracted teeth. Inclusion criteria included incisors with single canal, closed apex, and straight roots that had been extracted in the past 3 months for orthodontic treatment or due to periodontal disease. Exclusion criteria included the following: presence of any cracks, caries, resorption defects, decalcifications, or previous endodontic treatment.

### 2.1 Preparation and root canal treatment of the teeth

The teeth were cleaned with a periodontal curette and immersed in 10% formalin for 1 week for disinfection. The teeth were then stored in saline at room temperature (37°C) until the experiment. The teeth were decoronated perpendicular to the longitudinal axis of the root using a diamond bur and high‐speed handpiece under copious water irrigation such that the remaining root length was 15 mm. Next, #10 and #15 K‐files were passed through the canal orifice to ensure patency. In case of obstruction, RC‐Prep was used to achieve patency. Working length was determined by introducing a #10 K‐file (Maillefer‐Dentsply, Ballaigues, Switzerland) into the root canal until its tip was visible at the apical foramen; 1 mm was subtracted from this length to obtain the working length. The root canals were instrumented by ProTaper Universal rotary system to F5 (Dentsply Maillefer, Ballaigues, Switzerland) using the single‐length technique. After using each ProTaper file, the canal was rinsed with 1 ml of 2.5% NaOCl followed by 1 ml EDTA 17% for 60 s, after final irrigation the root canals were dried with paper points. The root canals were obturated using a #45 gutta‐percha point (Aria Dent, Tehran, Iran) as the master cone and #20 gutta‐percha accessory points (Aria Dent, Tehran, Iran) and AH26 sealer (Dentsply DeTrey, Konstanz, Germany). The teeth were radiographed to ensure adequate quality of root filling. Next, G‐Cavit temporary restorative material (3 M ESPE, Seefeld, Germany) was used to seal the orifice.

### 2.2 Placement of prefabricated posts

The obturated root canals were incubated at 37°C and 100% humidity for 1 week. Next, 10 mm of gutta‐percha was removed from each canal by #2 and #3 Gates‐Glidden drills (Dentsply, Maillefer, Switzerland) such that 5 mm of gutta‐percha remained at the apex to preserve the apical seal. Next, the drills present in the D.T. Light Post kit (D.T #3, Bisco Inc., Schaumburg, IL) were used for post space preparation. Eventually, the teeth were radiographed with a standard parallel angulation to ensure absence of gutta‐percha and sealer on the canal walls. The teeth were then randomly divided into four groups (*n* = 15) according to the final irrigation protocol:

- Group 1: (control group): 5 ml of 17% EDTA +5 ml of 5.25% NaOCl +5 ml of saline as final irrigant for 60 s.
- Group 2: Control group protocol +5 ml of 2% CHX as final irrigating solution for 60 s.
- Group 3: Control group protocol +5 ml of 5.25% NaOCl as final irrigating solution for 60 s.
- Group 4: Control group protocol +5 ml of QMix as final irrigating solution for 60 s.

The root canals in each group were dried with paper points. After rinsing the post space, the ED primer was applied on root canal walls with a microbrush. The primer was gently air‐thinned for 30 s, and the excess primer was removed by a paper point. Next, size 1 glass fiber posts (Reforpost, Angelus, Londrina, PR‐ Brazil) were cemented with Panavia F2 dual‐cure self‐adhesive resin cement (Kuraray, Tokyo, Japan). Dimensions of the fiber post size1 was as follow: diameter of apical post end: 0.70 mm, diameter of coronal post end: 1.3 mm with 6% taper and 20 mm length. For the cementation of the posts The A and B pastes present in the kit were mixed; 10 mm of the fiber post was placed in the canal and cemented to the canal walls. Self‐adhesive cement was applied on the surface of fiber post and it was placed in the canal space according to the manufacturer's instructions, and light‐cured for 20 s. After cementation to exposed dentin, the coronal part of the roots was covered with composite resin (Filtek Z250; 3 M ESPE, St. Paul, MN) and they were incubated at 37°C and 100% humidity for 1 week. The roots were radiographed after cementation of the posts.

### 2.3 Measuring the bond strength

The roots were sectioned by a high‐speed cutting machine (Mecatome T201A, Presi, Grenoble, France) under copious water irrigation perpendicular to the longitudinal axis of the root. Four sections with 1 ± 0.1 mm thickness (Elnaghy, 2014), were made of each root, two from the coronal third and two from the middle third of the root. The coronal sections were made at 2 mm distance from the coronal margin of the root. The thickness of sections was measured by a digital caliper (Mitutoyo, Tokyo, Japan). A universal testing machine (Z050; Zwick Roell, Ulm, Germany) was used to measure the push‐out bond strength of fiber post to root dentin. Load was applied apico‐coronally at a crosshead speed of 0.5 mm/min by a cylindrical stainless steel piston with 0.7 mm diameter to the center of fiber post in each section with no contact with the root dentinal wall. Maximum load applied right before debonding was measured and recorded in Newtons (N). The push‐out load was applied apico‐coronally and pushed the post towards the wider root cross‐section. Thus, there was no limitation for dislodgment of the post.

To calculate the push‐out bond strength in megapascals (MPa), the debonding force in Newtons (N) was divided by the cross‐sectional area using the formula P=F/A where A is the post‐dentin surface area, which was calculated using the formula below:

$$A=\pi \;\left(R+r\right){\left[h^{2}+{\left(R-r\right)}^{2}\right]}^{0.5}$$

In this formula, R indicates the coronal root canal radius, r indicates the apical root canal radius, and h indicates the thickness of each section. (Fundaoğlu Küçükekenci & Küçükekenci, 2019).

After measuring the push‐out bond strength, the mode of failure of each specimen was determined under a stereomicroscope (Olympus, SZ61, Olympus Optical Co., Tokyo, Japan) at x30 magnification. The failure mode was categorized as:

- Adhesive at the dentin‐cement interface.
- Adhesive at the post‐cement interface.
- Cohesive within the cement.
- Cohesive within the post.
- Mixed.

### 2.4 Statistical analysis

Data were analyzed using the SPSS version 22 via two‐way ANOVA and Tukey's post‐hoc test at *p* < 0.05 level of significance. Two‐way ANOVA was used to assess the effect of type of irrigating solution and location of cross‐section (coronal or middle third) on push‐out bond strength of fiber posts. Pairwise comparisons of irrigating solutions regarding the push‐out bond strength of fiber posts were carried out using the Tukey's test.

## 3 RESULTS

According to two‐way ANOVA, the effect of type of irrigating solution on push‐out bond strength of fiber posts to root dentin was significant (*p* = 0.002). However, the effect of location of cross‐section (middle or coronal third) on the push‐out bond strength was not significant (*p* = 0.054). The interaction effect of these two parameters on the push‐out bond strength was not significant (*p* = 0.658). In both the coronal and middle parts of the root, the maximum bond strength was noted in QMix group while the minimum bond strength was noted in 5.25% NaOCl group.

According to the Tukey's test, the bond strength of fiber post to root canal in 5.25% NaOCl group was significantly lower than that in the CHX (*p* < 0.02) and QMix (*p* < 0.004) groups but had no difference with saline group (*p* = 0.36).

Table 1 shows the mean push‐out bond strength in the coronal and middle thirds of the canal in the four groups in megapascals (MPa).

**Table 1:** Push‐out bond strength values in the coronal and middle thirds in the experimental groups in megapascals

Type of irrigant	Root zone	Mean	SD	Minimum	Maximum
Saline	Coronal	1.64	1.26	0.44	4.44
	Middle	2.5	2.06	0.27	6.51
CHX	Coronal	2.51	2.09	0.33	6.63
	Middle	2.69	1.81	0.52	5.99
NaOCl	Coronal	1.39	1.42	0.26	5.7
	Middle	1.55	1.49	0.27	6.73
QMix	Coronal	2.41	1.68	0.5	5.9
	Middle	3.22	1.92	0.69	6.76

Table 2 shows the frequency of the modes of failure in the four groups. According to the results, adhesive failure had the highest frequency in all groups.

**Table 2:** Percentage of different bond failure modes in the experimental groups

Irrigating solution	Mixed (number/percentage)	Cohesive in resin cement (number/percentage)	Cohesive in post	Adhesive at the resin‐dentin interface (number/percentage)	Adhesive at the cement‐post interface
Saline	16 (28.57)	9 (16.07)	0	31 (55.35)	0
CHX	13 (23.21)	5 (8.92)	0	38 (67.85)	0
NaOCl	10 (17.85)	7 (12.5)	0	39 (69.64)	0
QMix	23 (38.33)	0	0	37 (61.66)	0

Abbreviation: CHX, chlorhexidine.

**Figure 1:**
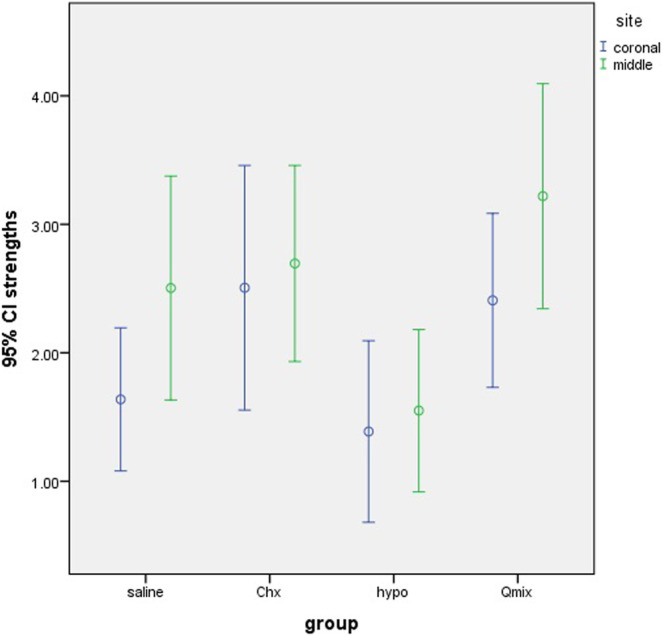
Error bar of the mean and 95% confidence interval of push‐out bond strength of fiber post to root dentin in the coronal and middle thirds

Figure 1 shows the error bar of the mean and 95% confidence interval of push‐out bond strength of fiber post to root dentin in the coronal and middle thirds.

## 4 DISCUSSION

Evidence shows that the bond strength to root canal wall depends on several factors (Wan et al., 2020) such as the root dentin properties, presence/absence of the smear layer, type and thickness of cement, type of bonding agent and its polymerization pattern, and the quality of endodontic treatment prior to post cementation with regard to the type and concentration of irrigating solution, sealer type, and so on (Niu et al., 2021). Similar to our findings, Gundugar et al. reported an increase in the bond strength of EndoSequence bioceramic sealer to root dentin following the use of QMix. They explained that this finding might be attributed to effective elimination of the smear layer by QMix (Gündoğar et al., 2018). Assessing the effect of different irrigating solutions, used for elimination of calcium hydroxide from the root canal system, on the bond strength of fiber‐reinforced composite posts revealed maximum bond strength in the QMix group, followed by MTAD and NaOCl/EDTA groups (Chaudhary et al., 2018). In 2013, Elnaghy et al. evaluated the effect of QMix on bond strength of glass fiber posts to root dentin. They reported that QMix and 17% EDTA +2% CHX yielded maximum bond strength to the entire root surface, compared with other irrigation protocols (Elnaghy, 2014).

According to the current results, the bond strength values were almost the same in CHX and QMix groups. High bond strength in the CHX group may be related to absorption of CHX by the root dentin and subsequently enhanced resin infiltration into dentinal tubules due to its non‐oxidizing property (Erdemir et al., 2004). CHX has a surfactant in its composition, which increases the surface energy of dentin and subsequently its wettability. Thus, it enhances the bond strength of fiber post to root dentin in use of resin cements (Hashem et al., 2009). CHX prevents or decreases the destruction of exposed collagen fibrils and preserves a stable hybrid layer, resulting in improved bond strength (Komori et al., 2009). It also prevents the host protease activity and preserves the morphological properties of the hybrid layer as such (Hebling et al., 2005).

According to the current results, minimum bond strength value was noted in NaOCl group. Sodium hypochlorite has extensive applications in endodontic treatment and its favorable efficacy for efficient debridement, root canal disinfection, lubrication, and tissue dissolution has been previously confirmed (Santos et al., 2006). The current results regarding the adverse effect of root canal irrigation with NaOCl on the bond strength of fiber post to root dentin are similar to the findings of a previous study (Elnaghy, 2014). Elnaghy et al. reported minimum bond strength to the entire root canal in the NaOCl group (Elnaghy, 2014).

The main reason for reduction of push‐out bond strength in the NaOCl group is production of free oxygen radicals by NaOCl, which serve as a barrier against the penetration and polymerization of adhesive resin (Hashem et al., 2009). Sodium hypochlorite oxidizes some of the constituents of the dentin matrix and leads to formation of free radicals derived from protein materials. These free radicals compete with the free radicals that are generated during the light‐curing process of resin, and resultantly, formation of polymer chains is stopped and the process of polymerization cannot be well completed (Lai et al., 2001; Morris et al., 2001). Also, evidence shows that root canal irrigation with sodium hypochlorite decreases the calcium and phosphorous contents, as well as the mechanical properties of dentin such as its modulus of elasticity, microhardness, and flexural strength. This can be related to decreased micromechanical reactions between the adhesive resin and dentin following the use of sodium hypochlorite (Santos et al., 2006).

According to the current results, the push‐out bond strength was not significantly different in the middle and coronal thirds of the root (*p* = 0.054), which was in agreement with the results of Gundugar et al (Gündoğar et al., 2018). However, the bond strength values in the middle third of the roots were slightly higher than those in the coronal third of the root in all specimens in this study. This finding may be attributed to the differences in number, volume, and direction of dentinal tubules in the root dentin (Onay et al., 2010; Zorba et al., 2010). Furthermore, chemical composition of the remaining sealer can affect the post bond strength to root dentin and contamination of dentinal walls with sealer and gutta‐percha can adversely affect this value (Dibaji et al., 2017b). Different parts of the root canal system may show variable bond strength values following the use of resin cement and different irrigation protocols. Such differences are attributed to the variable densities of dentin, technical sensitivity, difficult application of adhesive into the narrow post space, and limitations in light curing of the root canal system. Some studies have reported a reduction in bond strength from the coronal towards the apical region (Fundaoğlu Küçükekenci & Küçükekenci, 2019; Jowkar et al., 2020). However, in this study, the bond strength in all groups was slightly, but not significantly, higher in the middle third compared with the coronal third of the roots. Different bonding mechanisms (micromechanical or chemical) related to the use of dual‐cure resin cement may play a role in this respect (Jha & Jha, 2012). Another reason may be due to the light transfer through the fiber posts, improving the polymerization rate in the middle third. Also, according to Nemati et al, another reason may be the better adaptation of the post in the apical region and subsequently lower thickness of cement in this region, which would decrease the polymerization shrinkage (Nemati Anaraki & Sedighi, 2014). Dual‐cure adhesives are less dependent on light for polymerization. Thus, their application does not often cause a significant difference in bond strength in different parts of the root in comparison with light‐cure adhesives (Ebrahimi et al., 2014).

In this study, most failure modes were adhesive failure at the resin‐dentin interface which was similar to the results of Elnaghy et al, (Elnaghy, 2014) Bouillaguet et al, (Bouillaguet et al., 2003), and Afkhami et al. (Afkhami et al., 2018) However, in this study, similar to that of Abreu et al, the mode of failure in most specimens was adhesive at the resin cement‐dentin interface (Abreu et al., 2020). In the study by Bouillaguet et al, (Bouillaguet et al., 2003) higher frequency of adhesive failure at the resin cement‐root dentin interface was attributed to polymerization stresses in this region, due to the canal geometry. Optimal bonding of cement to root dentin is difficult to achieve (Bouillaguet et al., 2003). Thus, the C‐factor is high in this region, which would generate a high stress due to shrinkage of resin cement against the canal wall, that would compete with the bond strength at the adhesive interface (Carvalho et al., 2020). Moreover, according to Abreu et al. (2020), higher frequency of adhesive failure at the resin cement‐root dentin interface is due to chemical compatibility of the resin matrix of fiber post with cement and also adequate wetting of the post with the cement when bonding the fiber post margins.

## 5 CONCLUSION

Considering the maximum push‐out bond strength of fiber post to root dentin achieved following final root canal irrigation with QMix, it seems that it does not interfere with the bond strength of fiber posts to root dentin, and can be safely used for final irrigation of the root canal system.

## CONFLICT OF INTEREST

The authors declare no potential conflict of interest.

## AUTHOR CONTRIBUTION

Farzaneh Afkhami, Mona Sadegh and Maryam Amirmoezzi planned the study and research design and also data acquisition. Farzaneh Afkhami and Aidin Sooratgar performed the data analyze/interpretation and prepared the manuscript. All the authors read and approved the final manuscript.

## Data Availability

Data available on request from the authors.
